# Decision-making competence predicts domain-specific risk attitudes

**DOI:** 10.3389/fpsyg.2015.00540

**Published:** 2015-05-12

**Authors:** Joshua A. Weller, Andrea Ceschi, Caleb Randolph

**Affiliations:** ^1^School of Psychological Science, Oregon State UniversityCorvallis, OR, USA; ^2^Decision Research, EugeneOR, USA; ^3^Department of Philosophy, Education, and Psychology, University of VeronaVerona, Italy; ^4^Department of Psychology, Idaho State University, PocatelloID, USA

**Keywords:** decision-making competence, risk-taking, DOSPERT, domain-specific risk, individual differences, expected value, risk perception, risk-return model

## Abstract

Decision-making competence (DMC) reflects individual differences in rational responding across several classic behavioral decision-making tasks. Although it has been associated with real-world risk behavior, less is known about the degree to which DMC contributes to specific components of risk attitudes. Utilizing a psychological risk-return framework, we examined the associations between risk attitudes and DMC. Italian community residents (*n* = 804) completed an online DMC measure, using a subset of the original Adult-DMC battery. Participants also completed a self-reported risk attitude measure for three components of risk attitudes (risk-taking, risk perceptions, and expected benefits) across six risk domains. Overall, greater performance on the DMC component scales were inversely, albeit modestly, associated with risk-taking tendencies. Structural equation modeling results revealed that DMC was associated with lower perceived expected benefits for all domains. In contrast, its association with perceived risks was more domain-specific. These analyses also revealed stronger indirect effects for the DMC → expected benefits → risk-taking path than the DMC → perceived risk → risk-taking path, especially for behaviors that may be considered more maladaptive in nature. These results suggest that DMC performance differentially impacts specific components of risk attitudes, and may be more strongly related to the evaluation of expected value of a specific behavior.

## Introduction

Although choices that are based on sound decision-making principles may not always lead to the desired outcome, appropriately applying these rules can substantially increase the likelihood that positive outcomes will outnumber negative ones (Hastie and Dawes, 2010). Research has suggested that individual differences in the tendency to respond rationally to decisions are associated with numerous health and social outcomes (Parker and Fischhoff, 2005; Bruine de Bruin et al., 2007; Weller et al., 2015). Some researchers have referred to this construct as *decision-making competence* (DMC), which has been assessed by providing within-subjects versions of classical decision-making tasks, such as framing and over/under-confidence effects. Although past studies have suggested a link between DMC and real-world risk taking, little is known about the degree to which these tendencies are differentially associated with the perceptions of riskiness associated with an activity, compared to the perceived expected benefits for engaging in it. According to a psychological risk-return framework, these two dissociable, evaluative components have been shown to predict the likelihood that one will engage in a particular activity (Weber and Milliman, 1997; Weber et al., 2002). In the current study, we address this research question. Given the apparent domain-specificity of risk-taking behavior (e.g., Weber et al., 2002; Hanoch et al., 2006), we examined the degree to which individual differences in DMC are associated with risk taking, perceived risk, and perceived expected benefits across six domains. In contrast to prior studies that have reported associations between DMC and risk taking, the current study investigated how DMC relates to specific risk evaluations, which are believed to influence one’s risk-taking intentions, within the psychological risk-return model across a variety of domains. Guided by past research and theory, we propose that DMC may more strongly relate to the perceived expected benefits (i.e., involving tradeoffs between positive and negative consequences) of a particular activity than an activity’s perceived riskiness (i.e., involving perceptions of uncertainty).

Accumulating research suggests the existence of systematic individual differences in decision behavior and rational thought, a competence that may not be well-measured by traditional measures of general mental ability (e.g., Stanovich, 2009; Toplak et al., 2011). The tendency to respond rationally is believed to improve accuracy of choices, as the decision-maker more thoroughly defines the sample space of all possible events, seeks out and comprehends relevant information (see Hastie and Dawes, 2010). Subsequently, decision quality also hinges on effectively valuing and comparing options and filtering out irrelevant “noise” that is not central to the decision problem, such as incidental emotion or a particular decision frame (e.g., gain/loss). These tendencies have been conceptualized as a stable, trait-like construct, labeled “DMC.” In contrast to self-report measures that assess decision-making styles (e.g., Miller and Byrnes, 2001), DMC scores are based on objective performance with respect to either normative accuracy or the consistency of responses on several classic decision-making tasks. For instance, responding consistently to decision problems that involve the framing of otherwise equivalent objective information (e.g., lives saved/lost) would reflect consistency, whereas the ability to follow a decision rule to select the appropriate option given a multi-attribute matrix indicates accuracy of a response (Parker and Fischhoff, 2005). Supporting its construct validity, stable individual differences using different language versions of the DMC measures, have been observed, modified versions to accommodate a wide age range of participants (Bruine de Bruin et al., 2007; Del Missier et al., 2012; Mäntylä et al., 2012; Weller et al., 2012).

If DMC reflects the tendency to approach decisions from a perspective that stresses quality of the decision process rather than solely focusing on immediate outcomes, one might expect that greater DMC also will be associated with lower incidence of behaviors that may bear adverse long-term financial, social, or health consequences. Parker and Fischhoff (2005) found that lower DMC scores predicted higher incidence of delinquency, substance abuse, and risky sexual behaviors (e.g., number of sexual partners) in a sample of 18–19 years old males. With the revised adult version of the DMC measure (A-DMC), Bruine de Bruin et al. (2007) reported that lower DMC was associated with experiencing a greater number of negative decision outcomes (e.g., divorce). From a clinical perspective, Mäntylä et al. (2012) found that adults reporting features of Attention Deficit Hyperactivity Disorder, a disorder related to increased risk-taking behaviors such as substance use and sexual promiscuity (e.g., Barkley, 1998; Mannuzza and Klein, 2000), demonstrated poorer scores on the Applying Decision Rules scale of the A-DMC measure. Similar results also were observed for children. Weller et al. (2012) reported that greater DMC, using a child-friendly version of a subset of the original DMC measures, predicted a lower likelihood that a child was called to the principal’s office, and more likely that they reported following through on a challenging goal that was set for oneself. In a follow-up study, Weller et al. (2015) found that higher DMC scores at age 10–11 predicted fewer reported emotional, conduct, and peer problems two years later. Although these difficulties do not indicate greater risk taking directly, poor peer relations and conduct problems are often precursors to the initiation of health-risking behaviors such as substance use during adolescence (Clark et al., 2005; Tapert et al., 2005).

Although these results illuminate the associations beetween DMC and risk behavior, one must be mindful that risk behavior is a domain-specific, rathen than unidimensional construct (see Bromiley and Curley, 1992 for an early review). Weber et al. (2002) revitalized interest in the psychometric study of individual differences in domain-specific risk taking. To meet this end, they developed the Domain Specific Risk Taking scale (DOSPERT; see also, Blais and Weber, 2006). The DOSPERT assesses different component of risk attitudes (i.e., risk taking, risk perceptions, and perceived expected benefits) across six broad domains: Social (e.g., asking an employer for a raise), Recreational (e.g., skydiving), Investment (e.g., investing in a speculative stock), Gambling (e.g., betting a portion of income on a sporting event), Health/Safety (e.g., drinking too much alcohol at a party), and Ethical (e.g., cheating on a tax return) risks. Research has reinforced the external validity of the DOSPERT and the domain-specific approach to risk attitude. For instance, Hanoch et al. (2006) found that individuals who participated in extreme sports were more likely to accept risks associated with recreational risks than non-enthusiasts, but did not differ in their risk attitudes in other domains. Likewise, Markiewicz and Weber (2013) found that individuals reporting more favorable gambling risk attitudes were also more likely to engage in excessive stock trading. Similarly, Zimmerman et al. (2014) found that individual differences in ethical risk-taking scores predicted actual dishonest behavior in the laboratory (see also Gummerum et al., 2014, who examined this issue with currently- and previously incarcerated individuals). Finally, Rolison et al. (2014) found that age-related differences in risk attitudes demonstrated evidence of domain-specificity across the lifespan. Collectively, these findings suggest distinct patterns of risky choice behavior and specific correlates of each risk domain.

The DOSPERT self-report measure is theoretically derived from a psychological risk-return model, which was based on earlier financial models of risk-taking that state that risk-taking attitude is viewed a function of risk (i.e., variance) and return (i.e., expected value; Markowitz, 1959). Psychological risk-return models suggest that the propensity to engage in a risky activity can be conceptualized in terms of both the perceived riskiness involved with the activity and the expected benefits from engaging in an activity (Weber and Johnson, 2008). Additionally, although risks and benefits tend to be positively correlated in the world (or uncorrelated), they are inversely correlated in people’s minds (Slovic et al., 2004). Within this conceptualization, domain-specific differences in risk behavior emerge in how individuals evaluate and weight these two components (Weber et al., 2002). Research suggests that increases in the perceived riskiness of an activity will be associated with a lower likelihood that an individual will engage in the activity (i.e., less risk taking), whereas greater perceived benefits should result in greater risk taking (Finucane et al., 2000; Weber et al., 2002). Thus, risk behavior will vary across domains if there are differences in the magnitude of perceived risks and/or expected benefits (Weber et al., 2002).

With these points in mind, we predicted that DMC scores would be more strongly related to differences in the perceived expected benefits of the activity, compared to its perceived risks. When confronted with potential risky activities, individuals demonstrating greater DMC may be more likely to seek out, attend to, and evaluate a more complete set of information when making choices, subsequently leading to a more thorough evaluation of the tradeoffs between positive and negative consequences. Subsequently, these individuals may also more heavily weight the expected value of engaging in the activity, relative to the perceived riskiness. Preliminary evidence supports this hypothesis. Performance on complex decision tasks have suggested that expected value is related to executive function and inhibitory control, similar to patterns observed with DMC (e.g., Parker and Fischhoff, 2005; Henninger et al., 2010; Weller et al., 2012; Donati et al., 2014; Webb et al., 2014). Directly related to the current inquiry, Parker and Weller (accepted) observed that higher DMC scores were associated with a greater tendency to make choices based on expected value on a hypothetical behavioral risky decision-making task. Additionally, studies investigating the association between personality and decision-making have demonstrated that traits related to cognitive flexibility and deliberation, such as Openness to Experience and Conscientiousness, respectively, were more consistently associated with differences in expected benefits rather than the perceived risks of the activities (Weller and Tikir, 2011). In contrast, traits related to the experience of negative emotional states (e.g., Neuroticism) has been linked to heightened perceptions of risks, but not necessarily differences in perceived expected benefits (Butler and Matthews, 1987; Peters and Slovic, 1996; Chauvin et al., 2007; Weller and Tikir, 2011).

In the current study, we recruited an Italian community sample of participants, who were asked to complete Italian-language versions of both the A-DMC and the DOSPERT. We chose to include four component indices from the A-DMC: Resistance to Framing, Recognizing Social Norms, Applying Decision Rules, and Consistency in Risk Perception. These represented a sampling of two A-DMC indices that assessed rational responding via accuracy, and two indices that assessed consistency of responding. We did not include the Sunk Costs index because the relationship between Sunk Costs and the other A-DMC components are empirically unclear, with some studies suggesting a positive association with the other components (Bruine de Bruin et al., 2007), a negative relationship (Weller et al., 2012), inconsistent correlations (Del Missier et al., 2012), or no relationship at all (Parker and Fischhoff, 2005). We excluded the A-DMC Over/Underconfidence measure in order to reduce participant burden for this online study.

First, because we only used a subset of A-DMC indices, we conducted a confirmatory factor analysis (CFA) to determine whether a one-factor solution that resembled other DMC composite measures reasonably fit the observed data. Next, to test how DMC was differentially associated with risk perceptions, perceived expected benefits, and risk behavior (which we hereafter refer to as “risk taking”), we used a structural equation modeling (SEM) approach. This approach allowed for the examination of mediation effects; specifically, the degree to which risk perceptions and perceived benefits mediated the association between DMC and risk taking. Based on this model, we made four specific predictions:

H1: For all domains, risk perceptions will be inversely associated with risk taking, and perceived expected benefits will be positively associated with risk taking. Expected benefits and perceived risks will be inversely correlated.
H2: Greater performance on the DMC component measures will be associated with lower risk taking, especially for ethical, health/safety, and gambling risks. As an exploratory question, we tentatively predicted that DMC would be positively associated with social risk taking. Because of the scale’s limited scope content-wise, we made no explicit predictions regarding investment risk-taking.
H3: Across domains, greater DMC will be more strongly associated with expected benefits than perceived risks.
H4: The observed data from the four DMC indicators would fit reasonably well to a one-factor CFA solution.
H5: We predicted that expected benefits, and not perceived risks, would mediate the association between DMC and risk taking across all domains.

## Materials and Methods

### Participants

A third-party survey research firm sent 7044 invitations to an opt-in panel of Italian community residents to participate in this study. As a result of their participation, respondents collected points to get cash and other prizes. Of these, 921 subjects completed the entire survey. In the final sample, 50 participants were excluded who took less than 10 min to complete the entire survey (Median = 31.78 min). Additionally, 67 subjects were removed because they demonstrated no variance in scores across DOSPERT items, indicating careless responding, leaving a final *N* = 804.

The median age for the final sample was 35 years (58% female). For the participants who completed the study, 7.2% of participants did not possess a high school diploma, 52.2% had a high school diploma or equivalent, 21.4% received a bachelor’s degree, and 19.1% received an advanced college degree. This study was approved by the Ethical Review Committee at the University of Verona.

### Procedure

Participants were invited to take part in the survey via email, informing them that the survey was available. In the email, participants were provided with a hyperlink that directed them to the questionnaires. As part of a larger study, participants were asked to complete a battery of questionnaires, including the DOSPERT and A-DMC component measures. Additionally, participants completed a broad-based, dimensional personality measure and several hypothetical decision tasks that were unrelated to the current inquiry. Therefore, it will not be discussed further in this paper.

### Measures

#### Adult Decision Making Competence (A-DMC)

Individual differences in DMC were assessed by using the Italian version of the A-DMC, developed by Del Missier et al. (2012). The A-DMC components used were (see **Table 1** for descriptive statistics).

**Table 1:** Descriptive statistics and intercorrelations for the A-DMC.

	M (SD)	Skewness	Kurtosis	1	2	3	4
(1) Recognizing social norms	0.28 (0.30)	-0.47	-0.43	–			
(2) Decision rules	0.38 (0.20)	0.40	-0.40	**0.36**	–		
(3) Consistency in risk perception	0.65 (0.21)	-0.49	-0.23	**0.27**	**0.25**	–	
(4) Resistance to framing^a^	1.89 (0.50)	-0.60	0.33	0.09^∗^	**0.13**	0.06	–

*N* = 804. Correlations in bold are significant at *p* < 0.01.^∗^*p* < 0.05. ^a^Resistance to framing scores were reflected so that higher scores reflect greater performance.

##### Resistance to framing

This measure assessed the degree to which individual responses to decision problems were influenced by the framing of a decision problem, either in terms of risky choice framing (e.g., lives saved/lives lost; Tversky and Kahneman, 1981) or attribute framing (e.g., 95% success vs. 5% failure of a health prevention program; Linville et al., 1993). This measure consisted of 14 framing problems (seven of each type). Respondents scored their level of preference for a choice (i.e., either the risky or riskless option in the risky choice-framing task, or on an attribute, such as “effectiveness of program,” for the attribute framing items) on a 6-point Likert scale. Resistance to framing was measured by the mean absolute difference in response between the different frames of the same task. Scores were then reflected such that greater positive values related to greater resistance to framing. Cronbach’s α = 0.67.

##### Recognizing social norms

This measure assessed the understanding of social norms (16 items), and was adopted from Loeber (1989) and Jacobs et al. (1995). Participants first endorsed whether or not it is “sometimes OK” to engage in several behaviors that may be deemed undesirable (e.g., to steal under certain circumstances). Later in the assessment, respondents were asked to rate “out of 100 people your age,” how many would endorse each of the same behaviors. For each participant, performance was measured by the correlation between the actual endorsement rate of the behavior in the sample (out of 100%) and the estimated percentage of peers’ endorsements across the 16 behaviors. Because this component was calculated in this manner, reliability cannot be accurately estimated; however, Cronbach’s α = 0.76 for individual’s personal endorsement rates.

##### Applying decision rules

Respondents were asked to answer ten items that assessed their ability to follow a set of decision rules in order to make an accurate selection from five options in a multi-attribute matrix. Across the items, participants are asked to choose a DVD system that matches a hypothetical buyer’s search criteria (e.g., “Paolo wants to buy the DVD with the most attribute ratings that were above average.”). Each consumer chooses from a different set of five equally priced DVD players with varying ratings of picture quality, sound quality, programming options, and brand reliability (1 = *very low*; 5 = *very high*). Each problem had one correct response. Performance was represented as the percentage of correct responses, Cronbach’s α = 0.65.

##### Consistency in risk perception

This measure assessed the degree to which the participants followed probability rules. Respondents answered 10 items concerning the possibility of an event occurring to them within the next month. Later in the assessment, participants evaluated the possibility that the event would occur to them in the next two years. Correct responses were indicated by the probability of an event of occurring in the next month being no more than the probability of the same event occurring in the next two years. Performance was indicated by the number of probability-consistent responses made. Cronbach’s α = 0.57.

#### Domain-Specific Risk Taking

Participants completed an Italian version of the 40-item DOSPERT (Weber et al., 2002)^1^. The measure was adopted using a back-translation procedure with an independent bilingual translator. The DOSPERT measures individual differences in risk attitudes across six domains: *Health/Safety* (e.g., “Not wearing a helmet when riding a motorcycle”), *Ethical* (e.g., “Cheating on an exam”), *Recreational* (e.g., “Taking a skydiving class”), *Social* (e.g., “Approaching your boss to ask for a raise”), *Gambling* (e.g., “Playing in a high-money poker game”), and *Investment* (e.g., “Investing 5% of your annual income in a conservative stock”) risks. Participants assessed the likelihood that they would engage in a particular behavior (i.e., Risk taking), in addition to the activity’s perceived risks (Risk Perceptions) and the perceived expected benefits for engaging in the behavior (Expected Benefits). Following Weber et al. (2002), participants were asked to “please indicate your likelihood of engaging in each activity or behavior” on a 5-point Likert scale (1 = *Extremely unlikely;* 5 = *Extremely likely*). Risk perceptions were assessed by asking participants to indicate “how *risky* they perceived each of these activities (“For each of the following statements, please indicate *how risky* you perceive each situation”; 1 = *Not at all risky;* 5 = *Extremely risky*). Finally, we asked participants to rate the degree to which they would expect benefits from each activity (“For each of the following statements, please indicate *the benefits* you would obtain from each situation”; 1 = *No benefits at all;* 5 = *Great benefits*). We standardized the order of presentation for these assessments such that we first administered the Risk Taking scale, then the Risk Perception scale, and finally the Expected Benefits scale. DMC items as well as other scales unrelated to the current study were interspersed between the DOSPERT scales. **Table 2** shows the descriptive statistics of the DOSPERT scale.

**Table 2:** Descriptive statistics for the DOSPERT scale.

Scale	α	M (SD)	Skewness	Kurtosis
**Risk taking**
Health/safety	0.76	19.19 (6.09)	0.28	-0.44
Ethical	0.86	17.78 (6.87)	0.52	-0.40
Recreational	0.84	19.11 (7.12)	0.29	-0.76
Social	0.68	25.89 (5.04)	-0.46	0.59
Gambling	0.81	8.51 (3.94)	0.54	-0.67
Investment	0.83	10.42 (3.94)	0.02	-0.74
**Expected benefits**
Health/safety	0.84	29.54 (5.63)	-0.62	0.99
Ethical	0.86	28.27 (5.81)	-0.32	0.59
Recreational	0.82	28.35 (5.46)	-0.31	0.55
Social	0.82	21.39 (5.57)	0.21	-0.02
Gambling	0.72	14.79 (3.10)	-0.34	0.28
Investment	0.74	12.69 (3.11)	0.31	0.30
**Risk perceptions**
Health/safety	0.88	16.69 (6.83)	0.63	-0.37
Ethical	0.92	16.23 (7.29)	0.59	-0.75
Recreational	0.88	20.06 (7.45)	0.04	-0.86
Social	0.78	20.84 (5.76)	0.01	-0.01
Gambling	0.87	8.08 (3.83)	0.60	-0.67
Investment	0.88	9.21 (3.90)	0.10	-1.01

### Data-Analytic Strategy

First, we examined the zero-order correlations between the component DMC indices and domain-specific risk attitudes. For the primary analyses, SEM analyses were conducted to determine the degree to which expected benefits and perceived risks mediated the effects of DMC on domain specific risk taking. Then, CFA and SEM analyses were conducted with MPlus 7.2 (Muthén and Muthén, 1998–2012) software package. Parameters were estimated using the full-information maximum likelihood method, which uses all available information from all observations. Path parameters were freely estimated in this model. The variance of the latent variable was fixed at 1.0. We conducted 2000 bootstrap resamples in order to obtain *p*-values and reliable confidence intervals for the indirect effects (Shrout and Bolger, 2002; Edwards and Lambert, 2007).

**FIGURE 1:**
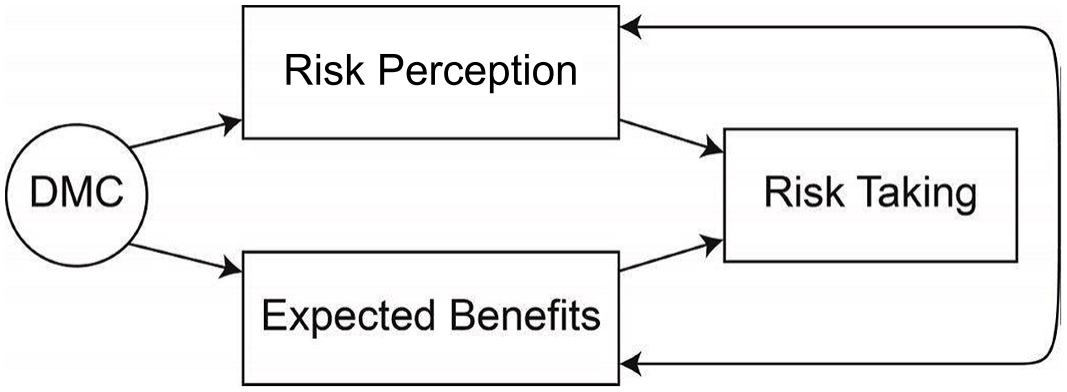
**Proposed mediation model**.

The measurement model for these analyses specified DMC as a one dimensional factor with four indicators. Because we used only a subset of DMC indices, we first conducted a CFA in the absence of other structural relations to test the degree to which our one-factor model fit the data. Then, a structural model was developed in line with the theoretical risk-return framework (see **Figure 1**). Specifically, this model specified that perceived risks and expected benefits fully mediated the relationship between DMC and risk taking. For model simplicity, perceived risks, expected benefits, and risk taking were treated as observed variables. We conducted six parallel SEM analyses, one for each risk domain. Model fit was evaluated using several established fit indices, including χ^2^ goodness of fit, root mean square error of approximation (RMSEA), standardized root mean square residual (SRMR), and comparative fit index (CFI; see Loehlin, 2004 for detailed explanation of fit indices).

To test for mediation, we compared three models: (a) the X (DMC) → Y (risk taking), (b) X → Y and X → M → Y partial mediation model, and (c) X → M → Y full-mediation model. The full mediation model was tested last determine if the removal of the direct path from DMC to risk taking provided a better model fit than the partial mediation model. We did not explicitly test via structural equation models, the X → M and M → Y models because the correlational analyses indicate significant relations, and henceforth suggest that the preliminary conditions for mediation were met (Baron and Kenny, 1986). Chi-square difference tests were conducted to determine whether the partial or full mediation model best fit the data.

## Results

### Associations between Risk Taking, Risk Perceptions, and Expected Benefits

As shown in **Table 3**, risk perceptions associated with a particular domain were inversely, and most strongly, related to risk taking in that domain. Similarly, expected benefits were positively, and most strongly, associated with risk taking in the corresponding domain. Additionally, with the exception of the Social risk domain, risk perceptions were inversely associated with expected benefits. Both risk perceptions and expected benefits were robust predictors of risk taking. With the exception of the social domain, these results support Hypothesis 1.

**Table 3:** Correlations between risk perceptions, expected benefits, and risk taking.

DOSPERT risk taking						
Scale	Health/safety	Ethical	Recreational	Social	Gambling	Investment
**Risk perceptions**
Health/safety	**-0.41**	-0.37	-0.24	-0.03	-0.30	-0.15
Ethical	-0.28	**-0.41**	-0.21	-0.12	-0.25	-0.16
Recreational	-0.28	-0.24	**-0.43**	-0.07	-0.21	-0.13
Social	0.02	0.08	0.06	**-0.29**	0.10	0.01
Gambling	-0.27	-0.30	-0.20	-0.06	**-0.45**	-0.20
Investment	-0.09	-0.11	-0.08	-0.16	-0.12	**-0.38**
**Expected benefits**
Health/safety	**0.57**	0.53	0.43	0.11	0.48	0.28
Ethical	0.47	**0.63**	0.38	0.08	0.49	0.31
Recreational	0.39	0.38	**0.70**	0.24	0.35	0.28
Social	0.35	0.37	0.32	**0.37**	0.33	0.28
Gambling	0.46	0.48	0.39	0.09	**0.66**	0.31
Investment	0.28	0.35	0.31	0.13	0.36	**0.58**

**DOSPERT Expected Benefits**						
**Risk perceptions**
Health/safety	**-0.40**	-0.37	-0.22	-0.16	-0.31	-0.23
Ethical	-0.28	**-0.36**	-0.25	-0.19	-0.23	-0.19
Recreational	-0.28	-0.26	**-0.42**	-0.15	-0.23	-0.20
Social	0.31	0.29	0.09	**0.09**	0.30	0.20
Gambling	-0.31	-0.32	-0.23	-0.19	**-0.41**	-0.25
Investment	-0.01	-0.07	-0.10	-0.06	-0.05	**-0.32**

*N* = 804. Within-domain correlations are in bold. Correlations greater than or equal to | 0.10| are significant at *p* < 0.01.

### Associations between Risk Taking and A-DMC Components

**Table 4** shows the correlations between DOSPERT risk taking, risk perception, expected benefit scales and the A-DMC components. Inspection of this table reveals several important trends. Overall, we found support for Hypothesis 2. We found that performance on the A-DMC components were significantly associated with self-reported risk taking for all domains, except Social risk taking. Specifically, lower DMC scores were related to more positive attitudes toward risk taking, especially for risky behaviors that are typically associated with lower inhibitory control, self-regulation, and greater sensation seeking. However, these direct associations were modest in effect size. Although these correlations were largely robust, we note two major exceptions. First, we found that DMC component scores were most weakly associated with risk taking in the investment domain. Scores on the individual DMC component measures were not associated with Investment risk taking, although these indices were inversely associated with both lower perceived risks and lower expected benefits, consistent in magnitude with the other DMC component measures. Second, the Resistance to Framing measure was not significantly associated with risk attitude in a systematic way. This finding is particularly notable, because half of the items in this scale were considered “risky choice” framing problems, in the tradition of the “Asian Disease” problem (Tversky and Kahneman, 1981). We conducted a follow-up correlational analysis between the domain-specific risk measures and both Resistance to Risky Choice Framing and Resistance to Attribute Framing, in order to rule out whether one form of framing attenuated these correlations. For both attribute and risky framing, we found no evidence that the aggregation of the two types of framing problems attenuated the overall Resistance to Framing correlations. The correlations between these framing subscales and risk attitudes were largely uniform, for risk-taking (Mean *r* = 0.03 and 0.03, for risky choice and attribute framing, respectively), risk-perceptions (Mean *r* = 0.05 and 0.04), and expected benefits (Mean *r* = -0.02 and 0.07).

**Table 4:** Correlations between A-DMC component scales and DOSPERT scales.

	Recognizing social norms	Decision rules	Consistency in RP	Resistance to framing
**Risk taking**
Health/safety	**-0.14**	**-0.11**	**-0.18**	-0.05
Ethical	**-0.18**	-0.09	**-0.17**	0.00
Recreational	**-0.17**	**-0.10**	**-0.18**	-0.03
Social	**0.13**	**0.16**	0.04	-0.08
Gambling	**-0.21**	**-0.11**	**-0.19**	-0.01
Investment	**-0.09**	0.01	-0.06	-0.03
**Risk perceptions**
Health/safety	**0.17**	**0.11**	**0.13**	-0.06
Ethical	0.04	-0.02	0.01	**-0.10**
Recreational	**0.14**	0.07	**0.11**	-0.02
Social	**-0.34**	**-0.31**	**-0.23**	-0.07
Gambling	**0.14**	0.09	**0.09**	-0.02
Investment	**-0.15**	**-0.17**	**-0.09**	-0.07
**Expected benefits**
Health/safety	**-0.31**	**-0.25**	**-0.26**	-0.03
Ethical	**-0.31**	**-0.21**	**-0.25**	-0.03
Recreational	**-0.11**	-0.06	**-0.14**	-0.01
Social	**-0.12**	-0.07	**-0.12**	-0.04
Gambling	**-0.31**	**-0.26**	**-0.24**	-0.03
Investment	**-0.20**	**-0.14**	**-0.17**	-0.02

*N = 804. Correlations in bold are significant at p < 0.01.*

Also, we found that the DMC components were more strongly associated with expected benefits, compared to perceived risks. To confirm these observations and provide further support for Hypothesis 3, we followed Steiger’s (1980) *z*-score procedure^2^ to test for differences between dependent correlations, comparing the correlation between DMC and risk perceptions and the correlation between DMC and expected benefits for each domain. We followed this procedure for three of the four component measures (Recognizing Social Norms, Applying Decision Rules, and Consistency in Risk Perception). For the Health/Safety, Ethical, and Gambling domains, we found that all of the nine comparisons were significant at *p* < 0.01. For the Investment and Recreational domains, we found no differences between perceived risks and expected benefits. In contrast, the Social domain showed the opposite pattern (i.e., *r_dmc-risk perception_* > *r _dmc-expected benefits_*). Therefore, we found mixed support for Hypothesis 3; the hypothesis was supported for the more “maladaptive” risks, but not for the more socially accepted risks. That is, individuals who scored lower on the DMC measure were more not only more likely to state that they would engage in risk behaviors like cheating on an exam or drinking too much at a party, but also their perceptions and expected benefits differed. Specifically, individuals with lower DMC scores typically saw less danger associated with these behaviors, as well as seeing more benefits associated with engaging in them.

### Measurement Model: Confirmatory Factor Analysis- DMC

Using CFA, we tested whether a one-factor DMC solution including the four DMC indicators fit our data. We expected that a unidimensional DMC latent construct would reasonably fit our data (Parker and Fischhoff, 2005; Bruine de Bruin et al., 2007; Weller et al., 2013). **Table 4** shows the path estimates and standard errors for the indicator variables. Supporting Hypothesis 4, this model yielded good fit to the data, suggesting that these indicators can be conceptualized as a latent DMC construct, χ^2^ (2) = 1.67, *p* = 0.45, CFI = 1.00, TLI = 1.00, RMSEA = 0.00, SRMR = 0.01. These fit indices were close to perfect due to the relatively low degrees of freedom. The standardized factor loadings all were significant at *p* <0.01. Reliability for the latent variable was calculated using McDonald’s omega, ω = 0.56.

### Structural Models Examining the Associations between DMC and Risk Attitude

**FIGURE 2:**
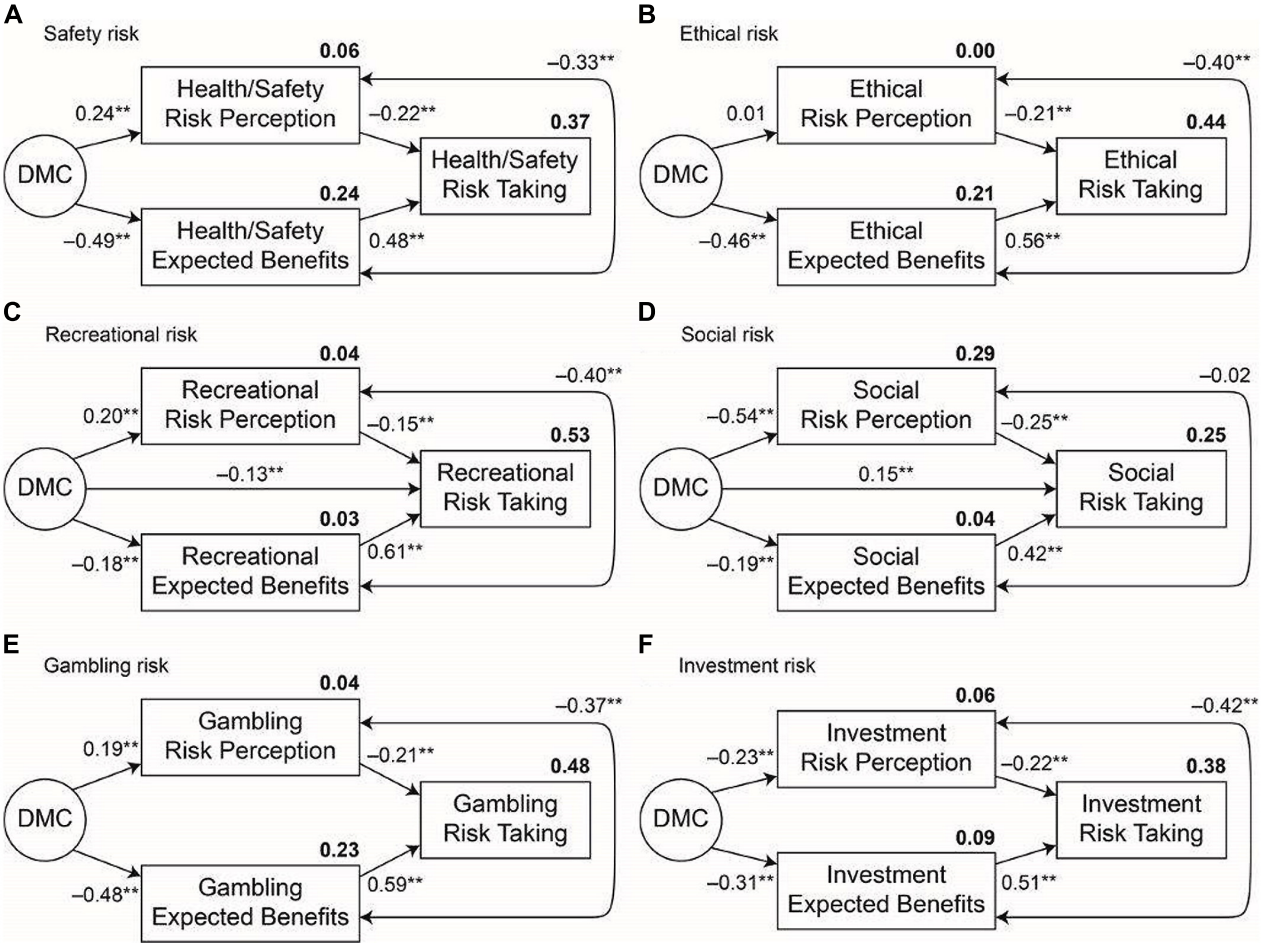
**Final structural equation models for each risk domain. (A–F)** Reflects the risk domain. ^∗∗^*p* < 0.01.

Next, we examined the degree to which risk perceptions and expected benefits mediated the associations between DMC and domain-specific risk taking. We conducted parallel SEM analyses for each risk domain. **Figure 2** shows the standardized parameter estimates for the final model of each analysis (see **Table 5** for fit indices). **Table 6** reports the specific standardized total and indirect effects for each domain.

**Table 5:** Standardized factor loadings for confirmatory factor analysis – DMC one factor solution.

			95% confidence interval	
Variable name	Parameter estimate	SE	Lower	Upper
Applying decision rules	0.59	0.05	0.5	0.69
Consistency in risk perception	0.43	0.04	0.34	0.51
Resistance to framing	0.18	0.05	0.09	0.27
Recognizing social norms	0.61	0.05	0.51	0.71

All parameter estimates significant at *p* < 0.001.

**Table 6:** Standardized effects (total and indirect), and confidence intervals of the indirect effects.

		Indirect effects on risk taking	
Risk domain	Total effects on risk taking	Expected benefits (95% CI)	Risk perception (95% CI)
Health/safety	-0.29**	-0.23** (-0.28, -0.18)	-0.05** (-0.08, -0.03)
Ethical	-0.26**	-0.26** (-0.32, -0.19)	0.00 (-0.02, 0.02)
Recreational	-0.26**	-0.11** (-0.17, -0.05)	-0.03** (-0.05, -0.01)
Social	0.20**	-0.08** (-0.12, -0.04)	0.13** (0.08, 0.19)
Gambling	-0.30**	-0.28** (-0.34, -0.22)	-0.04** (-0.06, -0.02)
Investment	-0.11**	-0.16** (–0.21, –0.11)	0.05** (0.02, 0.08)

**p < 0.01.

#### Direct Paths from Perceived Risks and Expected Benefits to Risk Taking

Consistent with past research, we found that risk perceptions and perceived benefits significantly contributed to the variance for all domains. Greater perceived risks in a particular domain were related to a lower likelihood of risk taking for those behaviors. Conversely, expected benefits were positively associated with risk taking. For all domains, the magnitude of the path between expected benefits and risk taking was stronger than the direct path from perceived risks to risk taking. Further, our results revealed inverse correlations between risk perceptions and perceived benefits, with the exception of the social domain which showed no correlation between these variables.

#### DMC and Risk Taking

##### Health/safety domain

As shown in **Figure 2** (Panel A), the final model revealed that DMC had a significant inverse path to expected benefits and a significant positive path to perceived risks. Although both the indirect effects from DMC to risk taking, via expected benefits and risk perceptions were significant, these effects were stronger for the DMC → expected benefits → risk taking path. The coefficient of the direct path from DMC to risk taking decreased from β = 0.25 in the X → Y regression model to β = 0.05 in the partial mediation model. The chi-square difference test confirmed that the full mediation model better fit our data, χ^2^_diff_ = 0.97, *p* = 0.34. Thirty-seven percent of the variance was explained in the final model.

##### Ethical risk domain

We found a similar pattern of results for the ethical risk domain. However, unlike the health/safety domain, the indirect effects were significant solely through the DMC → expected benefits → risk taking path. In fact, when accounting for expected benefits, DMC was not significantly associated with risk perceptions in the ethical domain (**Figure 2**, Panel B). The coefficient of the direct path from DMC to risk taking decreased from β = -0.26 in the X → Y regression model to β = 0.00 in the X → Y, X → M → Y model. Again, the chi-square difference test confirmed that the model with only indirect paths from DMC to risk taking better fit our data, χ^2^_diff_ = 0.02, *p* = 0.99. Forty-four percent of the variance was explained in the final model.

##### Recreational risk domain

In the final model for recreational risk taking, 52% of the variance was explained. DMC had a significant inverse path to expected benefits and a significant, but smaller in magnitude, positive path to perceived risks. Specifically, the coefficient of the direct path from DMC to risk taking decreased from β = -0.26 in the regression model without mediators to a reduced, but still significant, β = -0.13 in the partial mediation model (see **Figure 2**, Panel C). The chi-square difference test revealed that a partial mediation model provided a better fit for the data than the full mediation model, χ^2^_diff_ = 13.97, *p* < 0.001.

##### Social risk domain

In the final path model for the social risk domain (**Figure 2**, Panel D), 24% of the variance was explained. Different than the other risk domains, DMC had significant inverse paths to both expected benefits and perceived risks, with the latter path being stronger. Moreover, the indirect effects were significant for both indirect paths. The chi-square difference test revealed that the partially mediated model provided a better fit for the data χ^2^_diff_ = 7.35, *p* < 0.001. Specifically, the coefficient of the direct path from DMC to risk taking decreased from β = 0.20 in the regression model without mediators to β = 0.15, *p* < 0.01, in the partial mediation model.

##### Financial risk taking: a divergence between gambling and financial risks

With the DOSPERT measure, financial risk taking can be split into two subdomains: Gambling and Investment risks. Because gambling is typically seen as more of a problem risk behavior (e.g., Slutske, 2007), we predicted that the relationship between DMC and gambling risks would more closely resemble the results observed for the ethical and health/safety risk domains. In contrast, Investment risk scale items focus on more socially desirable risks that involve the potential for financial growth. Hence, in light of the observed results for Social Risk Taking, we tentatively speculated that higher DMC may be associated with greater Investment risk taking, and its relations with perceived risks and expected benefits may more closely resemble the pattern observed with social risks.

Consistent with our predictions, we found that Gambling risks were associated with the risk-return model in the same manner as ethical and health-safety risks (**Figure 2**, Panel E). As predicted, both indirect paths were significant, but the indirect effects for the DMC → expected benefits → risk-taking path were stronger than the indirect effects via risk perceptions. The DMC → risk-taking path reduced from β = -0.33 in the X → Y regression model to β = 0.02 in the partial mediation model. The chi-square difference test confirmed that the full mediation model better fit our data, χ^2^_diff_ = 0.18, *p* = 0.67. Forty-eight percent of the variance was explained with the full mediation model.

As shown in **Figure 2** (Panel F), we observed that DMC had significant direct paths to the mediator variables. Similar to social risk taking, greater DMC was related to lower perceived expected benefits and lower perceived risks associated with investment risk taking. Though we found significant indirect effects for investment risk taking, we did not find evidence to support any mediation in this domain; the parameter estimate for DMC in the X → Y regression model was β = -0.09, *p* = 0.075, suggesting no initial direct path, and hence, no mediation. Moreover, compared to the full mediation model, the X → Y, X → M → Y model did not improve model fit, χ^2^_diff_ = 0.11, *p* = 0.74, which suggests DMC only impacts investment risk taking indirectly. Thirty eight percent of the variance in risk taking was explained with the final model.

## Discussion

Although several studies previously have reported associations between DMC and risk taking, the current study is the first to investigate how the construct relates to specific risk evaluations within the psychological risk-return model that are believed to influence one’s risk behavior. Our results extend the literature in three main ways. First, consistent with our predictions and past research, we found that greater DMC was inversely associated with risk taking; this was especially the case for activities that are more likely to be deemed maladaptive in nature, such as ethical, health/safety, and gambling risks. Second, within the context of the risk-return model, our results suggest that DMC was more strongly related to expected benefits than risk perceptions, but only for risk behaviors that can be considered more problematic from a health, interpersonal, or financial perspective. We believe that this distinction may relate to a greater tendency to evaluate and appropriately weight positive and negative consequences, rather than a more affective evaluation about the perceived uncertainty of an activity. Third, the evaluation of potential expected benefits more strongly mediated the relationship between DMC and risk taking than did risk perceptions, with the exception of the social risk domain. These findings suggest that individual differences in DMC may not directly impact risk taking, but instead are associated with a preceding valuation process of potential hazards/activities.

Similar to past studies investigating this construct, the DMC components that we assessed reasonably converged to a one-factor model. This result reinforces that a latent variable signifying a broader cognitive competence may characterize performance on these tasks. Because we did not include the entire A-DMC measure, we cannot speak to the robustness of its factor structure extending beyond these four indicators. As a result, we must temper our conclusions, not only as they apply to the unidimensionality of the construct, but as they apply to the excluded component tasks. However, the pattern of correlations that we observed are consistent with other DMC studies. More specifically, the observed inter-item correlation matrix strongly resembles that reported in the only other known use of the Italian version of the A-DMC (Del Missier et al., 2012). We especially note potential associations between Over/Underconfidence with risk attitudes that were observed previously (Parker and Fischhoff, 2005; Bruine de Bruin et al., 2007), and traits related to real-world risk behaviors (e.g., narcissism; Campbell et al., 2004).

Somewhat surprising, our results revealed that Resistance to Framing was not associated with risk-taking behaviors. Notably, Parker and Fischhoff’s (2005) original study found significant correlations between their framing measure and risk behaviors. We offer two potential explanations for these inconsistencies. First, these two studies differed in the operationalization of decision outcomes which may have impacted this divergence. Specifically, Parker and Fischhoff (2005) assessed reports of actual risky behavior that is typically considered to be health-risking (i.e., substance use and sexual behavior in isolation). In contrast, the current study assessed risk behavior intentions. Though we cannot rule this point out, Bruine de Bruin et al. (2007) also did not observe an association between resistance to framing and self-reported negative decision outcomes, similar to the current study. Because the Bruine de Bruin et al. (2007) study measured Resistance to Framing in the same way as the current method, we suggest that another potential reason for these discrepancies may be due to the breadth of the Resistance to Framing measures used in the Y-DMC and A-DMC. The Y-DMC Resistance to Framing scale adopts a broader perspective of framing, including an inter-temporal choice item, for instance, in addition to traditional risky choice and attribute framing items. Given that delay discounting has been linked with impulsiveness, this may have influenced the results (e.g., Lawyer et al., 2010). In contrast, the A-DMC measure takes a more conceptually narrow approach to framing, focusing solely on attribute and risky choice framing. Although further examination of this issue falls outside the scope of the current study, we feel that this DMC component may benefit from further development in order to better establish content validity. Potential inclusions may involve the problems included in the original Y-DMC (e.g., Fischhoff, 1993; Shafir, 1993), but also other forms of framing problems such as ones that are believed to access more verbatim-level processing as proposed by Fuzzy Trace Theory (Reyna and Brainerd, 1995; Kühberger and Tanner, 2010).

With respect to the issue of domain-specific risk taking, the current study not only yielded an Italian-language version of the 40-item DOSPERT scale, but also it further reinforced the construct validity of the DOSPERT measure. With the exception of the Social risk domain, we found that expected benefits positively predicted risk taking within each domain, whereas perceived riskiness of an activity inversely predicted risk behaviors. Additionally, we found that perceived risks and expected benefits were inversely correlated with one another in all domains. Further, we found that individual DMC scores were differentially associated with perceived risks and expected benefits across the six risk domains, supporting the assertion that risk behaviors are domain-specific.

Moreover, our observed results resonate with the theoretical perspective of risk-return models. We found that DMC was more strongly associated with perceived expected benefits, which is theoretically tied to the evaluation of expected value, than perceived risks, which is associated with the evaluation of the variance of choice options (e.g., Markowitz, 1959; Weber and Milliman, 1997). These two types of evaluations may arise from two distinct processes: a more affective pathway focusing on evaluations of *risk/uncertainty,* and a more deliberative pathway that involves tradeoff assessments between potential positive and negative consequences. In fact, the directions that are used for the elicitation of perceived risk and expected benefits are likely to facilitate different processing modes. Specifically, perceived riskiness of an activity by asking the participant to rate “*your gut level assessment of how risky* each situation or behavior is,” potentially facilitating more experiential processing (Weber et al., 2002). In contrast, expected benefits are elicited by asking participants to indicate “*the benefits* you would obtain from engaging in the following activities.” In this light, basing decisions on the expected benefits, or expected value, can be viewed as a more deliberative process due to the necessity for accurately evaluating and integrating net outcome magnitude and the probability that each potential consequence will be realized. Because both DMC and sensitivity to expected value are both purported to recruit deliberative thinking and cognitive control processes (Bruine de Bruin et al., 2007; Henninger et al., 2010; Del Missier et al., 2012; Weller et al., 2012, 2015; Donati et al., 2014), individuals demonstrating greater DMC may be more likely to more thoroughly evaluate and appropriately weight both potential positive and negative consequences. In contrast, risk perceptions are commonly believed to be strongly affect-laden and experiential in nature (Slovic et al., 2004). Although it extends outside of the scope of this study, we speculate that the evaluation of variance (i.e., risk) may involve less taxing computational processes, and thus, may be less related to DMC, and more broadly, executive control function. We are conducting follow-up studies to further test these hypotheses.

With respect to the measurement of DMC, we call upon researchers to progressively move toward standardized assessments across studies in order to increase interpretability of results across studies. We acknowledge that rational responding on other types of decision-making paradigms, which are not included in the DMC battery, may also constitute “competent decision-making.” Although other tasks may indeed serve as an indicator for DMC, our findings reinforce the notion that the current battery represents a sample of behaviors/performance that may reflects a higher-order latent variable indicating individual differences in normative responding. We encourage such future psychometric inquiry. Consideration of other related skills can improve understanding of the construct’s phenotypic structure by better determining its dimensionality, and widening its nomological network. Of course, one potential challenge to the expansion and further testing of this construct, especially with respect to developing DMC batteries in other cultures, is to maintain the integrity of the component measures while simultaneously being sensitive to the fact that individuals from other cultures may have a different knowledge base (e.g., in the case of overconfidence), or prevailing social norms (e.g., as in the case of recognizing social norms). A second challenge relates to more practical concerns. Addition of measures to the DMC battery will lengthen assessment times, which may not be feasible in some circumstances. In this regard, item-response theory approaches to streamline measures ultimately may prove to be useful, both in terms of time-saving and improving the reliability of the measures.

At a broad level, these results may also have implications for interventions that are designed to improve decision-making. If decision-making skills can be taught (Baron and Brown, 1991; Dhami et al., 2012), one direct implication may be that risk behaviors may attenuated, either by decision-making education. For instance, Jacobson et al. (2012) found improvements in DMC scores by integrating a decision-education curriculum into a U.S. History high school history course. Additionally, developing computerized decision aids may assist in several regards. First, it may help the individual better understand his/her own preferences and long-term goals. Second, these systems may effectively guide the individuals through the decision problem to arrive at a choice that are consistent with those aims, preserving cognitive effort in the decision maker when facing risky choices (see Diehl et al., 2003). Aside from direct interventions, interventions designed to improve self-regulatory capacities may also reduce risk behaviors, as well as decision-making tendencies. As an example, Weller et al. (in press) found that maltreated adolescent girls who were randomly assigned to an early foster-care intervention (at age 11) designed to improve emotion regulation, self-control, and goal-setting (Chamberlain et al., 2006) made more normative decisions (i.e., on the basis of the expected value of a gamble) on a risky decision-making task (administered at age 16 years) than maltreated girls who received foster care services as usual. Although maximal effects would likely occur using a combination of these techniques, studies such as these offer a promise that decision-making skills are indeed malleable.

## Conclusion

This study extends past research by demonstrating the relationship between risk taking and DMC, and potential pathways by which DMC may influence risk evaluations. Our findings reinforce the domain-specificity of risk, and how individual differences in DMC may relate specifically to risky activities across different domain. Moreover, this study demonstrated that DMC is preferentially associated with perceived expected benefits, compared to risk perceptions. This finding supports the assertion that DMC impacts risk assessment through a more reasoned, deliberative pathway, as opposed to a more affective one. We hope that these results may ultimately lead to improved risk communication efforts and bolster interventions designed to abate risky behaviors.

## Author Contributions

JW had primary responsibility for design, data analysis, and overall project direction. AC was responsible for data acquisition and development of the online questionnaire. CR conducted preliminary analyses and presented a portion of these data at the 2013 Western Psychological Association annual conference. All authors contributed to the analysis, interpretation, and writing for the research described here.

## Conflict of Interest Statement

The authors declare that the research was conducted in the absence of any commercial or financial relationships that could be construed as a potential conflict of interest.

## Footnotes

1 During this process, we changed the content of two questions. First, the item in the Original DOSPERT, “Betting a day’s income at the horse races” was changed to “betting a day’s income at a slot machine” because horse racing is uncommon in Italy. Second, we changed the original question, “eating high-cholesterol foods” to “regularly eating foods that are fried and unhealthy foods.”

2 This approach derives a *Z*-score to be tested between the two correlations of interest (*r_jk_–r_jh_*), while accounting for the correlation, *r*_kh_.
